# Flavorubredoxin, a Candidate Trigger Related to Thrombotic Thrombocytopenic Purpura: Screening of the Complete Genome of a *Salmonella enterica* Serovar Typhimurium Isolate From an AIDS Case

**DOI:** 10.3389/fcimb.2022.864087

**Published:** 2022-06-10

**Authors:** Zhouhan Wang, Hao Xu, Beiqing Gu, Yanqi Jin, Tianyuan Wang, Jindi Ma, Yingfeng Lu, Xiaopeng Yu, Beiwen Zheng, Yimin Zhang

**Affiliations:** ^1^ State Key Laboratory for Diagnosis and Treatment of Infectious Diseases, National Clinical Research Center for Infectious Diseases, Collaborative Innovation Center for Diagnosis and Treatment of Infectious Diseases, The First Affiliated Hospital, College of Medicine, Zhejiang University, Hangzhou, China; ^2^ Department of Clinical Laboratory, The First Affiliated Hospital, College of Medicine, Zhejiang University, Haining, China; ^3^ Department of Infectious Diseases, The First Affiliated Hospital, College of Medicine, Zhejiang University, Haining, China

**Keywords:** *Salmonella*, HIV, thrombotic thrombocytopenic purpura, ADAMTS13, flavorubredoxin

## Abstract

Thrombotic thrombocytopenic purpura (TTP) is one of the two classic thrombotic microangiopathy (TMA) diseases which could be induced by infections. To the best of our knowledge, this is the first report of an acquired immunodeficiency syndrome (AIDS) patient with acquired TTP induced by infection with *Salmonella enterica* serovar Typhimurium (hereafter, *S*. Typhimurium) isolate, *S*. Typhimurium_zhang, which was confirmed by serology and genetic taxonomy. The literature review identified 17 TMA-related genes encoding the candidate triggers, which were searched in the annotated genome sequence of *S*. Typhimurium_zhang. Anaerobic nitric oxide reductase flavorubredoxin (FlRd), encoded by *norV* which is related to another TMA, haemolytic uraemic syndrome (HUS), was found in *S*. Typhimurium_zhang. Basic local alignment search tool (BLAST) analysis revealed that *norV* and FlRd in *S*. Typhimurium_zhang, as well as eight *S*. Typhimurium type strains, have high identity with HUS-related *Escherichia coli* O157:H7 strain TW14359. Similar results were obtained from the BLAST analysis of 73 *S*. *enterica* isolates for congenital TTP which was also previously reported to be triggered by *S*. *enterica*. Phylogenetic analysis and amino acid sequence alignment revealed that FlRd was functional and highly conservative on 69 Enterobacteriaceae, including *S*. Typimurium_zhang and TW14359. In brief, we found *norV* in the genome of a *S*. Typhimurium clinical isolate that induced TTP in an AIDS patient. FlRd, the protein encoded by *norV*, probably triggered the TTP and was highly conservative, functional, and widespread in *S*. *enterica* and Enterobacteriaceae. More *in vitro* and *in vivo* studies are required to confirm our findings and determine the underlying mechanism.

## Introduction

Thrombotic thrombocytopenic purpura (TTP) is one of two classic thrombotic microangiopathy (TMA) diseases induced by significantly reduced activity of metalloproteinase with thrombospondin type 1 motif, member 13 (ADAMTS13). The pathophysiological mechanisms underlying TTP mainly include the formation of ultra-large von Willebrand factor (vWF) in circulation, leading to spontaneous platelet aggregation (Moake, 2002; Lämmle and George, 2004; Sadler et al., 2004; Kremer Hovinga et al., 2017). The clinical features of TTP include systematic platelet agglutination, ischemia of fetal organs (particularly the brain, heart, gastrointestinal tract, and kidneys), severe thrombocytopenia, and intravascular hemolysis (Moake, 2002; Lämmle and George, 2004; Sadler et al., 2004; Kremer Hovinga et al., 2017). Infection is one of several causes of TTP (Kremer Hovinga et al., 2017).

Previously, infection-induced TTP has been reported with shiga toxin-producing *Escherichia coli*, hepatitis A virus, dengue virus, influenza virus, and SARS-CoV-2 (Thrombotic Thrombocytopenic Purpura Associated With Escherichia Coli O157:H7–Washington, 1986; Bitzan and Zieg, 2018; Albiol et al., 2020; Gogireddy et al., 2020; Beaulieu et al., 2021; Montgomery et al., 2021). Previous research has revealed that congenital TTP (cTTP) can be triggered by *S*. *enterica* infection (Wendt et al., 2021). To the best of our knowledge, acquired TTP induced by *S*. *enterica* serovar Typhimurium (hereafter, *S*. Typhimurium) has not been reported previously. Herein, we describe a patient with acquired immunodeficiency syndrome (AIDS) who developed TTP after infection. The pathogen was isolated from the peripheral blood, identified by clinical and genomic analysis as *S*. Typhimurium; and was designated *S*. Typhimurium_zhang. Then, the previous literature was searched to identify the possible bacterial triggers of TTP. Seventeen genes encoding for the candidate triggers were identified. The factors were searched in the annotated genome sequence of the isolated *S*. Typhimurium_zhang. The conservation degree and distribution in *S*. *enterica* and Enterobacteriaceae of the candidate triggers identified in *S*. Typhimurium_zhang were analyzed using genomic taxonomic methods and protein alignment.

## Materials and Methods

### Ethical Approval

This study was approved (2021IIT145) by the Ethics Committee of the First Affiliated Hospital, College of Medicine, Zhejiang University (Hangzhou, China), following the Declaration of Helsinki.

### Clinical and Laboratory Data Collection

The clinical information, including history of present illness, past history, physical examination, laboratory tests, radiographic examination, and treatment were obtained from the medical records.

Peripheral blood sample was obtained from bilateral elbow veins on the fifth day of admission. The blood was collected in two vials from each side and immediately sent for culture of aerobic and anaerobic bacteria. Blood culture was performed using BacTALERT 3D blood culture system (bioMérieux, Marcy l’Etoile, France) following the manufacturer’s instructions. For samples with positive results, the liquid in the vials was inoculated onto blood agar, chocolate, MacConkey, and fungal chromogenic plates at 35°C and 5% CO_2_ for 18–24 hours. The morphologically different colonies were sub-cultured on separate agar plates. The isolated bacteria were identified using the VITEK^®^ MS microbial mass spectrometry identification system (bioMérieux). Then, VITEK2^®^ COMPACT automatic identification and antibiotic sensitivity analysis system was used to verify the bacterial identification and perform antibiotic sensitivity analysis. For *S*. *enterica*, agglutination test using the corresponding agglutinating serum was performed to identify the species of the isolate. The isolate was named *S*. Typhimurium_zhang. Then, individual colonies were inoculated in broth tubes. After multiplication for 4–6 h, 10% glycerin broth was added to the tubes and stored at –80°C.

### Literature Search for Genes Encoding the TMA Triggers

Search terms related to TTP, hemolytic uremic syndrome (HUS), and TMA, as well as their synonyms, were used to search for English articles published up to November 2021 that focused on the link between the aforementioned diseases and their bacterial triggering factors, including genes and proteins. The search results were manually screened for errors.

### Whole-Genome Sequencing (WGS), Annotation, and Triggers Searching of *S.* Typhimurium_zhang

The frozen bacteria in glycerol lyophilized tubes were incubated on Mueller-Hinton (MHA) plates overnight at 37°C. A single colony was incubated in 2 mL Luria-Bertani (LB) broth at 200 rpm and 37°C overnight. We inoculated 1 mL bacterial solution in 100 mL LB broth at 200 rpm and 37°C for 6 h. The shaken bacterial solution was collected into 50 mL centrifuge tubes and centrifuged at 5000 g for 15 min, and the supernatant was discarded. The bacteria were suspended in phosphate-buffered saline (PBS) and transferred to a 1.5 mL Eppendorf (EP) tube. The EP tubes were centrifuged at 5000 g for 5 min and the supernatant was discarded for DNA extraction and sequencing. Genomic DNA was extracted using a commercial kit (Gentra Puregene Yeast/Bacteria kits; Qiagen, Hilden, Germany) following the manufacturer’s instructions. The Single Molecule, Real-Time (SMRT) sequencing library was constructed using SMRT bell TM Template kit (version 1.0). A next-generation sequencing (NGS) library was constructed using NEBNext^®^ Ultra™DNA Library Prep Kit for Illumina. The WGS data were processed using the PacBio Sequel platform and Illumina NovaSeq PE150 at the Beijing Novogene Bioinformatics Technology Co., Ltd (Beijing, China). The reads were assembled using SPAdes (version 3.9.1). The annotation of the genome sequence was performed using RAST (https://rast.nmpdr.org/). The genes encoding the candidate triggers obtained from the previously published studies were searched in the annotation result of *S*. Typhimurium_zhang. Antimicrobial resistance (AMR) genes were identified using the ResFinder database (https://cge.food.dtu.dk/services/ResFinder/) and the virulence factors were identified using the Virulence Factor Database (VFDB, http://www.mgc.ac.cn/VFs/search_VFs.htm).

### Genomic Identification of *S.* Typhimurium_zhang

To identify the species and genus of *S*. Typhimurium_zhang, all eight type strains of *S*. Typhimurium were downloaded from the American Type Culture Collection (ATCC) and National Collection of Type Cultures (NCTC), regardless of their completeness, and compared to the genomic sequence of *S*. Typhimurium_zhang. Average nucleotide identity blast (ANIb) analysis was performed using pyani (https://github.com/widdowquinn/pyani). The ANIb data for each strain were visualized using heatmaps.

### Identity Analysis of Genes and the Encoded Triggers on 83 *S*. *enterica* Strains

The complete sequences of TW14359, based on the search result of *S*. Typhimurium_zhang, were obtained from the National Center for Biotechnology Information (NCBI, https://www.ncbi.nlm.nih.gov/). All eight type strains of *S*. Typhimurium were downloaded from ATCC and NCTC, regardless of its completeness, and 73 *S*. *enterica* strains with complete genomes were downloaded from NCBI. The genome sequence of each strain was annotated by RAST. The identity of genes and their encoded TMA triggers present in *S*. Typhimurium_zhang were evaluated in these 83 *S*. *enterica* strains using BLAST (https://blast.ncbi.nlm.nih.gov/Blast.cgi).

### Phylogenetic Analysis of the Triggers in 69 Enterobacteriaceae Strains, Including *S*. Typhimurium_zhang and TW14359

In total, 69 non-repetitive Enterobacteriaceae type strains were downloaded from NCBI. The genome sequences of 69 Enterobacteriaceae strains were annotated by RAST and searched for genes encoding the candidate triggers present in *S*. Typhimurium_zhang. The amino acid sequences of the candidate triggers in 69 Enterobacteriaceae strains were downloaded from NCBI. Phylogenetic analysis of the candidate triggers on 69 Enterobacteriaceae strains, including *S*. Typhimurium_zhang and TW14359, was conducted using Molecular Evolutionary Genetics Analysis across Computing Platforms (MEGAX; version 10.1.7) software and were visualized using the Interactive Tree of Life (iTOL, https://itol.embl.de).

### Amino Acid Sequence Alignment With Crystal Structure Reference

According to the phylogenetic analysis, the one closest and the two farthest strains to *S*. Typhimurium_zhang, carrying the candidate triggers on the phylogenetic tree, were included in the amino acid sequence alignment. Meanwhile, the candidate triggers were uploaded to SWISS-MODEL (https://swissmodel.expasy.org/) and used to search for templates. Both known crystal structure templates for the protein were included in the amino acid sequence alignment and the protein closest to the candidate trigger on *S*. Typhimurium_zhang was used as the crystal structure reference in the alignment. The sequence of the candidate triggers in *S*. Typhimurium_zhang and reference strain related to TMA as well as sequences mentioned above were downloaded and aligned using CLUSTALW (https://www.genome.jp/tools-bin/clustalw) and ESpript 3.0 (Robert and Gouet, 2014) (https://espript.ibcp.fr/ESPript/cgi-bin/ESPript.cgi).

### Data Availability and Parameters of Bioinformatic Procedures

The complete genome data of the isolated *S*. Typhimurium_zhang has been uploaded to GenBank (accession number: CP090304; https://www.ncbi.nlm.nih.gov/nuccore/CP090304). The bioinformatics procedures described previously were performed using the default settings.

## Results

### Case Presentation

A 35-year-old man presented with black stool, gingival bleeding, and nausea (without vomiting) for 1 day. He passed 10 dilute black stools without any obvious cause 1 day before the admission. The laboratory tests at admission showed an extremely reduced platelet count, mildly abnormal renal function, and normal liver function. He had normal vital signs, with scattered skin petechiae, and oozing of blood from the gums. He had no underlying diseases, venereal disease exposure, or significant marital or family history.

**Figure 1:**
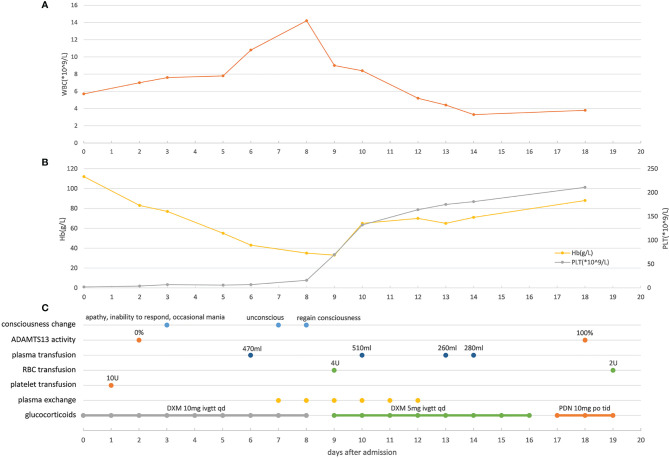
Main clinical manifestations, test results, and treatment related to TTP **(A)** Changes of white blood cell counts in peripheral blood of the patient during hospitalization. **(B)** Changes of hemoglobin and platelet in peripheral blood of the patient during hospitalization. **(C)** Changes of the patient’s consciousness, ADAMTS13 activity and timepoints of applying plasma transfusion, red blood cell transfusion, platelet transfusion, plasma exchange and glucocorticoids. WBC, white blood cell; Hb, hemoglobin; PLT, platelet; RBC, red blood cell. ADAMTS13, metalloprotease with thrombospondin type I motif, member 13; DXM, dexamethasone; PDN, prednisone.

**Figure 2:**
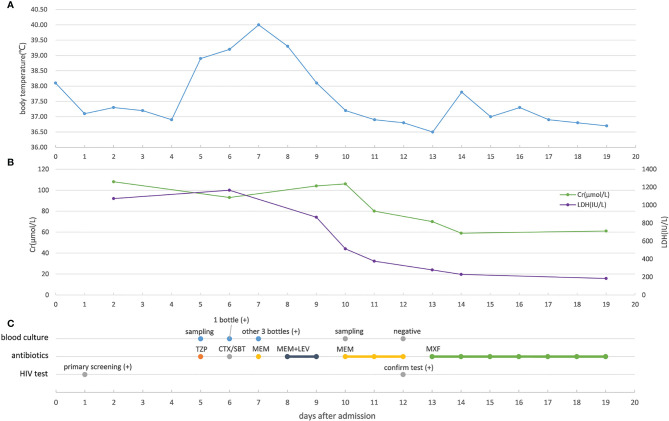
Changes in key indicators related to severity and anti-infection treatment **(A)** Changes of the patient’s body temperature during hospitalization. **(B)** Changes of creatinine and lactate dehydrogenase in the patient’s peripheral blood during hospitalization. **(C)** Blood culture result, antibiotic treatment, and HIV test result of the patient. Cr, creatinine; LDH,actate dehydrogenase; TZP, piperacillin/tazobactam; CTX/SBT, cefotaxime/sulbactam; MEM, meropenem; LEV, levofloxacin; MXF, moxifloxacin.

The human immunodeficiency virus (HIV) antibody was detected in a colloidal gold assay screening test and later confirmed by Western blotting. The baseline laboratory test results showed normal blood coagulation function, extremely low platelet count (2 × 10^9^/L), and mildly reduced hemoglobin (112 g/L). The lymphocytes showed significant reduction in CD4+ T cells (166.1 cells/µL). Blood samples were taken to analyze the activity of metalloproteinase with thrombospondin type I motif, member 13 (ADAMTS13) enzyme before initiation of plasma exchange and glucocorticoid therapy; the results showed 0% activity. Tests for hepatitis B surface antigen (HBsAg), hepatitis C (HCV) antibody, syphilis spirochete antibody, cytomegalovirus (CMV) IgM, and Epstein-Barr virus (EBV) IgM were negative. Identical Gram-negative bacteria were detected from blood cultures in all four tubes. The bacteria were identified as *S*. Typhimurium using agglutination tests and *via* microbial mass spectrometry 6 days after admission to the hospital. Ultrasound revealed deep venous thrombosis in the right lower limb. Routine examination of bone marrow and biopsy indicated decreased levels of megakaryocytes, without abnormal cells. The disease course and laboratory test results of the patient are shown in **Figures 1** and **2**. The patient presented with thrombocytopenia, microangiopathic hemolytic anemia, fever, mild renal dysfunction, and significant neuropsychiatric symptoms. Based on the absent ADAMTS13 activity, the patient was diagnosed with TTP according to the guidelines of Haemostasis and Thrombosis Task Force of the British Committee for Standards in Hematology (BCSH) (Scully et al., 2012). Our patient had reversible ADAMTS13 activity deficiency, indicating that he had acquired TTP.

The patient received one platelet transfusion, four plasma transfusions, two red blood cell transfusions, and five sessions of plasma exchange. The treatment also included the use of antipyretics, intravenous midazolam for sedation, glucocorticoids for immune suppression, antibiotics (including moxifloxacin) for treating infections, and heparin and rivaroxaban for anticoagulation. The patient did not receive antiviral therapy including antiretroviral therapy (ART). After treatment, the patient’s body temperature and laboratory test results returned to normal, and his symptoms improved significantly. The patient was discharged on day 19 and ART was continued at home.

### Clinical Identification of *S.* Typhimurium_zhang

The patient’s blood culture returned a positive result at 24 h. Similar gray-white colonies were detected on blood agar, chocolate, and MacConkey plates, whereas no growth was detected on the fungal chromogenic plate. The colonies detected on the blood agar, chocolate, and MacConkey plates were identified as *Salmonella* by the VITEK**^®^** MS microbial mass spectrometry identification system. VITEK2^®^ COMPACT automatic identification and antibiotic sensitivity analysis system confirmed the identification. Antibiotic sensitivity analysis was performed using VITEK2^®^ COMPACT and showed that the bacteria were sensitive to ampicillin, amoxicillin-potassium clavulanate, piperacillin/tazobactam, ceftriaxone, cefepime, ciprofloxacin, levofloxacin, imipenem, ertapenem, and compound sulfamethoxazole. Agglutination test results showed that the isolate agglutinated with *Salmonella* O4 serum but not with other O serum. The isolate also agglutinated with Hi, H1, and H2. Based on these results, the isolate was determined to be *S*. Typhimurium.

### WGS, Genomic Identification, and Annotation of *S*. Typhimurium_zhang

**Figure 3:**
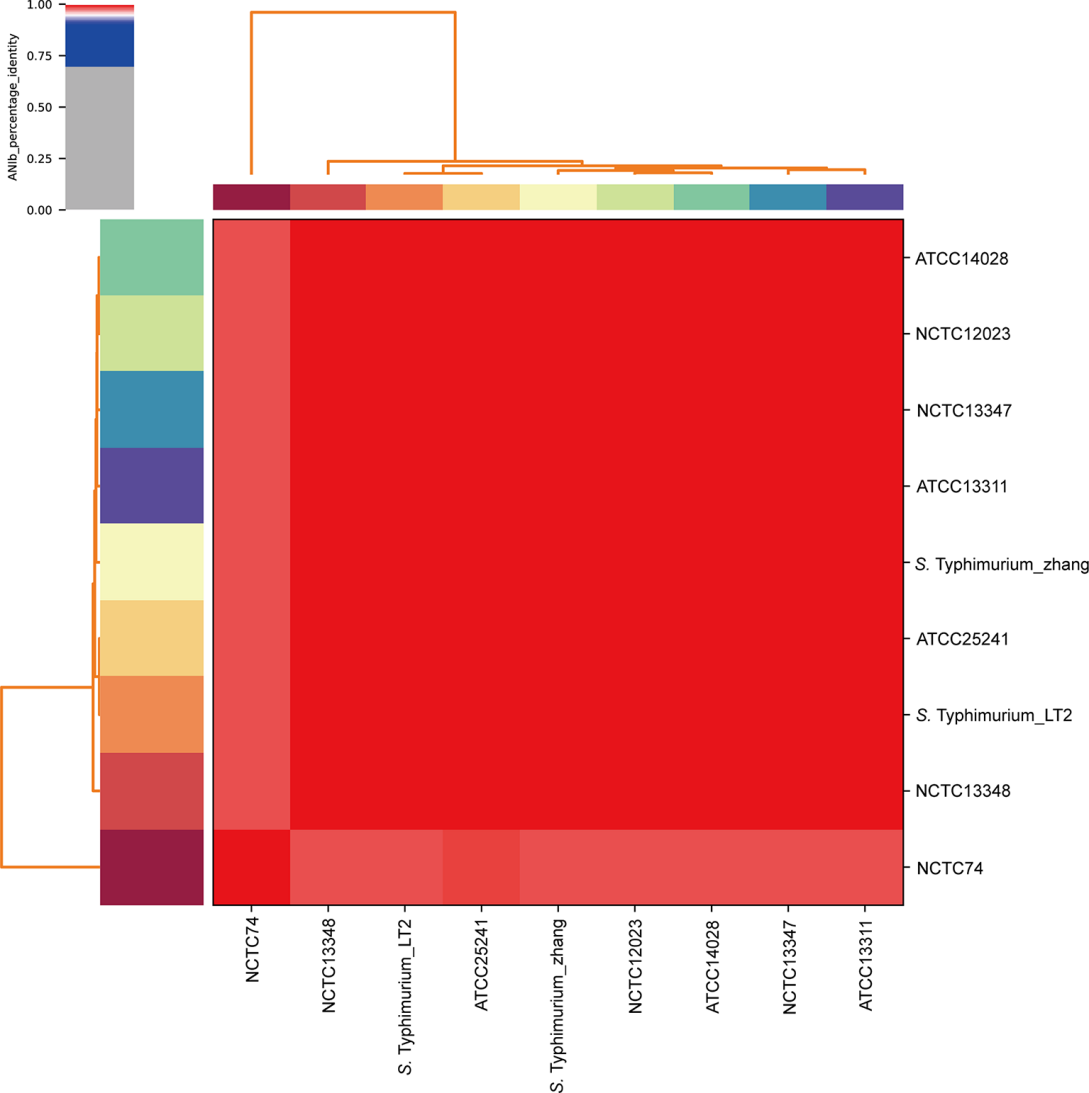
ANIb analysis of *S.* Typhimurium_zhang and eight *S.* Typhimurium type strains Eight *S.* Typhimurium type strains (ATCC14028, NCTC12023, NCTC13347, ATCC13311, ATCC25241, Typhimurium_LT2, NCTC13348, NCTC74) were downloaded and compared with the genomic sequence of *S.* Typhimurium_zhang. ANIb analysis was completed using pyani with default settings and visualized by heatmaps. ANIb, average nucleotide identity blast.

The WGS revealed that *S*. Typhimurium_zhang had 4857450 bps with 52.2% GC content. To confirm the species of *S*. Typhimurium_zhang, ANIb analysis was performed with eight type strains. The ANIb analysis showed that >95% identities matched between the eight type strains and *S*. Typhimurium_zhang, which confirmed that the pathogenic bacteria isolated from the peripheral blood were *S*. Typhimurium (Altschul et al., 1997; Konstantinidis and Tiedje, 2005; Richter and Rosselló-Móra, 2009) (**Figure 3**). The genes, virulence factors, and AMR genes of *S*. Typhimurium_zhang are presented in **Supplementary Tables 1–3**.

### Genes Encoding the Candidate Triggers Related to TMA

No previous reports were identified regarding the genes encoding the candidate triggers associated with TTP. The genes encoding the candidate triggers associated with HUS included *stx2* (Ethelberg et al., 2004; Pianciola et al., 2014; Alconcher et al., 2021), *eae* (Ethelberg et al., 2004; Pianciola et al., 2014; Alconcher et al., 2021), *iha* (Naseer et al., 2017), *IpfA* (Naseer et al., 2017), *ehxA* (Ethelberg et al., 2004; Naseer et al., 2017; Alconcher et al., 2021), *stcE* (Naseer et al., 2017), *rfb*_O157_ (Alconcher et al., 2021), *fliC*_H7_ (Alconcher et al., 2021), *nleB* (Cadona et al., 2020), *espP* (Buvens and Piérard, 2012), *vtx2* (Buvens and Piérard, 2012), *sen* (Buvens and Piérard, 2012), *nle* (Buvens and Piérard, 2012; Naseer et al., 2017), *efa* (Buvens and Piérard, 2012), *saa* (Zweifel et al., 2004), *norV* (Kulasekara et al., 2009), and *aggR* (Scheutz, 2014).

### Genes Encoding Candidate Triggers Present on *S*. Typhimurium_zhang

The genes encoding the candidate triggers identified from the previous literature were searched in the annotation result of S. Typhimurium_zhang. The results showed that S. Typhimurium_zhang carried *norV*, a gene encoding anaerobic nitric oxide reductase flavorubredoxin (FlRd). *NorV* was related to the increased rate of developing HUS as shown in a previous study (Kulasekara et al., 2009).

### Identification of *norV* Gene and Its Encoded Protein FlRd in 83 *S*. *enterica* Strains

The annotation results showed that all 83 *S*. *enterica* strains carried *norV*, including *S*. Typhimurium_zhang, TW14359, and 8 *S*. Typhimurium type strains. The BLAST results showed that *norV* and FlRd in *S*. Typhimurium_zhang, HUS-related *E*. *coli* O157:H7 strain TW14359, and eight *S*. Typhimurium type strains had high identity (**Table 1**). *NorV* and its encoded protein FlRd in 73 *S*. *enterica* strains presented higher identity compared to *norV* and FlRd in *S*. Typhimurium_zhang (**Table 2**).

**Table 1:** Identity of *norV* and encoded FlRd on *S.* Typhimurium_zhang, eight *S.* Typhimurium type strains and *E. coli* O157:H7 isolate TW14359 based on BLAST.

Microbial strain name	GenBank assembly accession	ref-*S.* Thyphimurium_zhang						ref-TW14359				
		*norV* (gene)			FlRd (protein)			*norV* (gene)			FlRd (protein)	
		Coverage (%)	Identity (%)		Coverage (%)	Identity (%)		Coverage (%)	Identity (%)		Coverage (%)	Identity (%)
*E. coli* TW14359	GCA_000022225.1	100	84.02		100	92.90		100	100		100	100
NCTC74	GCA_015565735.1	100	99.93		100	97.91		100	83.97		100	93.32
ATCC13311	GCA_000743055.1	100	99.93		100	99.79		100	84.02		100	93.11
ATCC25241	GCA_019990605.1	100	99.93		100	99.79		100	84.02		100	93.11
NCTC13348	GCA_900706765.1	100	99.93		100	99.79		100	84.02		100	93.11
*S.* Typhimurium_LT2	GCF_000006945.2	100	99.93		100	99.79		100	84.02		100	93.11
*S.* Typhimurium_zhang	GCA_021399255.1	100	100		100	100		100	84.02		100	92.90
ATCC14028	GCA_003864015.1	100	100		100	100		100	84.02		100	92.90
NCTC12023	GCA_900457195.1	100	100		100	100		100	84.02		100	92.90
NCTC13347	GCA_900456925.1	100	99.93		100	100		100	84.02		100	92.90

FlRd stands for anaerobic nitric oxide reductase flavorubredoxin. BLAST stands for Basic Local Alignment Search Tool.

**Table 2:** Identity of *norV* and FlRd on 73 *S. enterica* strains based on BLAST.

		*S.* Typhimurium_zhang				
		*norV* (gene)			FlRd (protein)	
Microbial strain name	GenBank assembly accession	Coverage(%)	Identities(%)		Coverage(%)	Identities(%)
***Salmonella enterica* subsp. *enterica* serovar Typhimurium**	GCA_003864015.1	100	100		100	100
***Salmonella enterica* subsp. *enterica* serovar Typhimurium**	GCA_016864495.1	100	100		100	100
***Salmonella enterica* subsp. *enterica* serovar Typhimurium**	GCA_000743055.1	100	99.93		100	99.79
***Salmonella enterica* subsp. *enterica***	GCA_900635565.1	100	98.96		60	99.31
***Salmonella enterica* subsp. *enterica* serovar Thompson str. ATCC 8391**	GCA_000486365.2	100	99.24		100	98.75
**Salmonella enterica subsp. *enterica* serovar Sloterdijk str. ATCC 15791**	GCA_000486445.2	100	99.03		100	98.75
***Salmonella enterica* subsp. *enterica* serovar Thompson**	GCA_900475825.1	100	99.24		100	98.75
***Salmonella enterica* subsp. *enterica* serovar Thompson**	GCA_900478375.1	100	99.24		100	98.75
***Salmonella enterica* subsp. *enterica* serovar Goldcoast**	GCA_900635695.1	100	99.24		100	98.75
***Salmonella enterica* subsp. *enterica***	GCA_900636155.1	100	99.24		100	98.75
***Salmonella enterica* subsp. *enterica* serovar Inverness str. ATCC 10720**	GCA_000487155.2	100	99.24		100	98.54
***Salmonella enterica* subsp. *enterica* serovar Enteritidis**	GCA_003031995.1	100	99.1		100	98.54
***Salmonella enterica* subsp. *enterica* serovar Java**	GCA_900086565.1	100	98.82		100	98.54
***Salmonella enterica* subsp. *enterica* serovar Heidelberg**	GCA_900478405.1	100	99.1		100	98.54
***Salmonella enterica* subsp. *enterica* serovar Daytona**	GCA_900635085.1	100	98.96		100	98.54
***Salmonella enterica* subsp. *enterica***	GCA_900635515.1	100	98.68		100	98.54
***Salmonella enterica* subsp. *enterica* serovar Havana**	GCA_900635855.1	100	98.68		100	98.54
***Salmonella enterica* subsp. *enterica* serovar Pullorum str. ATCC 9120**	GCA_000330485.2	100	98.96		100	98.33
***Salmonella enterica* subsp. *enterica* serovar Senftenberg str. ATCC 43845**	GCA_000486525.2	100	98.89		100	98.33
***Salmonella enterica* subsp. *enterica* serovar Rubislaw str. ATCC 10717**	GCA_000486585.2	100	98.4		100	98.33
***Salmonella enterica* subsp. *enterica* serovar Albany str. ATCC 51960**	GCA_000487515.2	100	98.26		100	98.33
***Salmonella enterica* subsp. *enterica* serovar Abaetetuba str. ATCC 35640**	GCA_000487915.2	100	98.33		100	98.33
***Salmonella enterica* subsp. *enterica* serovar Dublin str. ATCC 39184**	GCA_001953035.1	100	98.89		100	98.33
***Salmonella enterica* subsp. *enterica* serovar Stanley**	GCA_900475965.1	100	98.89		100	98.33
***Salmonella enterica* subsp. *enterica* serovar Aberdeen**	GCA_900477885.1	100	98.82		100	98.33
***Salmonella enterica* subsp. *enterica* serovar Infantis**	GCA_900478235.1	100	98.89		100	98.33
***Salmonella enterica* subsp. *enterica* serovar Pomona str. ATCC 10729**	GCA_000240905.3	100	98.12		100	98.12
***Salmonella enterica* subsp. *enterica* serovar Tennessee**	GCA_003031875.1	100	98.61		100	98.12
***Salmonella enterica* subsp. *enterica* serovar Senftenberg**	GCA_003864035.1	100	98.82		100	98.12
***Salmonella enterica* subsp. *enterica* serovar Montevideo**	GCA_003864055.1	100	98.61		100	98.12
***Salmonella enterica* subsp. *enterica* serovar Carmel**	GCA_900477895.1	100	98.54		100	98.12
***Salmonella enterica* subsp. *enterica* serovar Sundsvall**	GCA_900477905.1	100	98.12		100	98.12
***Salmonella enterica* subsp. *enterica* serovar Senftenberg**	GCA_900478065.1	100	98.82		100	98.12
***Salmonella enterica* subsp. *enterica***	GCA_900635555.1	100	98.54		100	98.12
***Salmonella enterica* subsp. *enterica***	GCA_900636165.1	100	99.03		100	98.12
***Salmonella enterica* subsp. *enterica* serovar Minnesota str. ATCC 49284**	GCA_000486855.2	100	98.4		100	97.91
***Salmonella enterica* subsp. *enterica* serovar Mbandaka str. ATCC 51958**	GCA_000486915.2	100	98.89		100	97.91
***Salmonella enterica* subsp. *enterica* serovar Chester str. ATCC 11997**	GCA_000487255.2	100	98.47		100	97.91
***Salmonella enterica* subsp. *enterica* serovar Choleraesuis str. ATCC 10708**	GCA_000487295.3	100	98.4		100	97.91
***Salmonella enterica* subsp. *enterica* serovar Anatum str. ATCC BAA-1592**	GCA_000487575.2	100	98.54		100	97.91
***Salmonella enterica* subsp. *enterica* serovar Senftenberg**	GCA_001457675.1	100	98.4		100	97.91
***Salmonella enterica* subsp. *enterica* serovar Typhimurium**	GCA_015565735.1	100	98.26		100	97.91
***Salmonella enterica* subsp. *enterica* serovar Bredeney**	GCA_900478205.1	100	98.33		100	97.91
***Salmonella enterica* subsp. *enterica* serovar Menston**	GCA_900478345.1	100	98.33		100	97.91
***Salmonella enterica* subsp. *enterica***	GCA_900635525.1	100	98.82		100	97.91
***Salmonella enterica* subsp. *enterica* serovar Sanjuan**	GCA_900635545.1	100	98.68		100	97.91
***Salmonella enterica* subsp. *enterica***	GCA_900635615.1	100	98.26		100	97.91
***Salmonella enterica* subsp. *enterica***	GCA_900635645.1	100	99.03		100	97.91
***Salmonella enterica* subsp. *enterica* serovar Paratyphi A str. ATCC 9150**	GCA_000011885.1	100	98.33		100	97.70
***Salmonella enterica* subsp. *enterica* serovar Paratyphi A str. ATCC 11511**	GCA_000486725.2	100	98.33		100	97.70
***Salmonella enterica* subsp. *enterica* serovar Panama str. ATCC 7378**	GCA_000486765.2	100	98.33		100	97.70
***Salmonella enterica* subsp. *enterica* serovar Poona str. ATCC BAA-1673**	GCA_000493295.2	100	98.4		100	97.70
***Salmonella enterica* subsp. *enterica s*erovar Give**	GCA_900477925.1	100	98.19		100	97.70
***Salmonella enterica* subsp. *salamae* serovar Greenside**	GCA_900478195.1	100	96.32		100	97.70
***Salmonella enterica* subsp. *salamae***	GCA_900478225.1	100	97.08		100	97.70
***Salmonella enterica* subsp. *enterica* serovar Poona**	GCA_900478385.1	100	98.4		100	97.70
***Salmonella enterica***	GCA_900478435.1	100	97.22		100	97.70
***Salmonella enterica* subsp. *arizonae***	GCA_900635595.1	100	97.29		100	97.70
***Salmonella enterica* subsp. *arizonae***	GCA_900635075.1	100	97.16		43	97.61
***Salmonella enterica***	GCA_900475895.1	100	97.08		100	97.49
***Salmonella enterica* subsp. *salamae***	GCA_900477985.1	100	96.94		100	97.49
***Salmonella enterica* subsp. *arizonae***	GCA_900478105.1	100	97.01		100	97.49
***Salmonella enterica* subsp. *enterica***	GCA_900635575.1	100	98.4		100	97.49
***Salmonella enterica* subsp. *enterica***	GCA_900635585.1	100	98.12		100	97.49
***Salmonella enterica* subsp. *enterica* serovar Florida**	GCA_900477995.1	100	98.4		100	97.29
***Salmonella enterica* subsp. *salamae***	GCA_900635535.1	100	96.94		100	97.29
***Salmonella enterica* subsp. *houtenae***	GCA_900635725.1	100	97.01		100	97.29
***Salmonella enterica* subsp. *houtenae* serovar Houten**	GCA_900478215.1	100	97.01		100	97.08
***Salmonella enterica* subsp. *arizonae***	GCA_900635675.1	100	96.32		100	97.08
***Salmonella enterica* subsp. *diarizonae***	GCA_900478155.1	100	96.25		100	96.87
***Salmonella enterica* subsp. *enterica***	GCA_900635605.1	100	98.89		91	96.34
***Salmonella enterica* subsp. *salamae***	GCA_900635655.1	100	96.18		95	95.85
***Salmonella enterica* subsp. *enterica***	GCA_900635865.1	100	98.41		99	95.61

FlRd stands for anaerobic nitric oxide reductase flavorubredoxin. BLAST stands for Basic Local Alignment Search Tool.

### Phylogenetic Analysis of FlRd in Enterobacteriaceae

**Figure 4:**
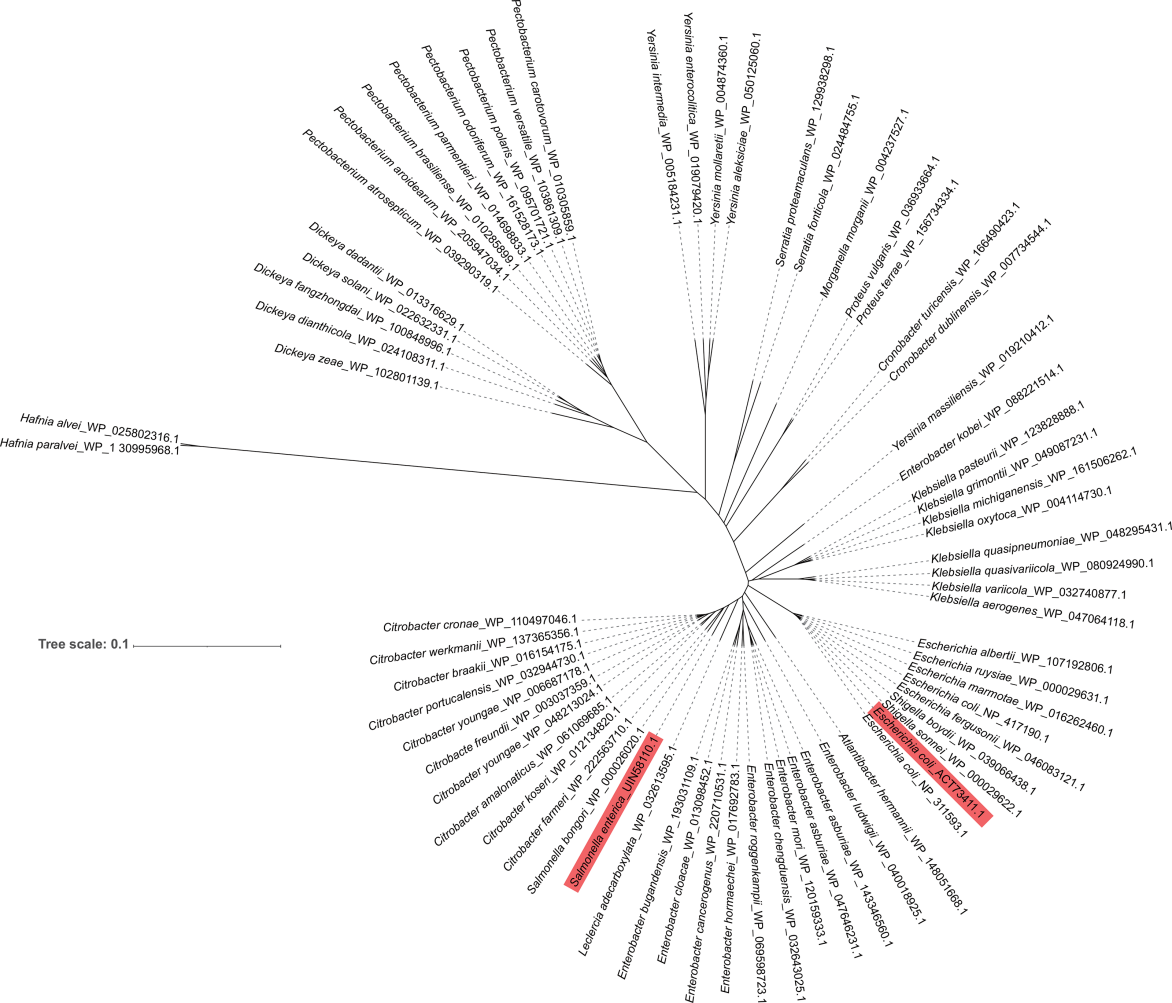
Phylogenetic analysis of FlRd on 69 Enterobacteriaceae strains including *S.* Typhimurium_zhang and TW14359 Amino acid sequence of FlRd on 69 *Enterobacteriaceae* strains including *S.* Typhimurium_zhang and TW14359 were downloaded from NCBI and included in the phylogenetic analysis. FlRd=anaerobic nitric oxide reductase flavorubredoxin. FlRd of *S.* Typhimurium_zhang (Salmonella enterica_UIN58110.1) and TW14359 (*Escherichia coli*_ACT73411.1) were highlighted in the phylogenetic tree.

The search of NCBI for Enterobacteriaceae that carry FlRd identified 69 strains. Phylogenetic analysis of these 69 Enterobacteriaceae was performed. Among them, Salmonella_bongori_WP_000026020.1 was the closest to FlRd in *S*. Typhimurium_zhang, whereas Hafnia_alvei_WP_025802316.1 and Hafnia_paralvei_WP_130335968.1 were the farthest from FlRd in *S*. Typhimurium_zhang **(** **Figure 4** **)**.

### Amino Acid Sequence Alignment Using the Crystal Structure for Reference

According to the phylogenetic analysis, Salmonella_bongori_WP_000026020.1, Hafnia_alvei_WP_025802316.1, and Hafnia_paralvei_WP_130335968.1 were included in the amino acid sequence alignment. A SWISS-MODEL search revealed that 5lmc.2 and 6etb.1 were the crystal structure templates for FlRd. The template search of TW14359 revealed similar results. Therefore, 5lmc.2 and 6etb.1 were included in the amino acid sequence alignment. Among them, 5lmc.2 had the highest similarity and was used as the crystal structure reference in the alignment. The FlRd in *S*. Typhimurium_zhang and TW14359 was also included in the alignment. The results indicated a high degree of similarity in the sequences, indicating that FlRd in *S*. Typhimurium_zhang was highly likely to be functional (**Supplementary Figure 1**).

## Discussion

TMA is a group of microvascular occlusive diseases associated with pregnancy, drugs, infection, transplantation, and cancer (Bayer et al., 2019). It mainly includes TTP and HUS, characterized by thrombocytopenia, microangiopathic hemolytic anemia, and fever. It is usually accompanied by mild renal abnormalities and significant neuropsychiatric symptoms. In comparison, renal abnormalities are prominent in HUS (Moake, 2002). The pathophysiological mechanisms underlying HUS and TTP have many similarities, such as complement mutations, increased nucleosome levels, and microangiopathic hemolysis.

Due to its high mortality and complex pathophysiology, TTP has received significant attention worldwide. However, its etiology and pathogenesis have not been fully elucidated. Previous studies have shown that isolated ADAMTS-13 deficiency may not cause TTP (Desch and Motto, 2007). Endothelial activation induced by infection or drugs may be the “second hit” in the pathogenesis of TTP (Kremer Hovinga et al., 2017). In animal models, ADAMTS-13 knockout mice did not develop TTP spontaneously and required stimulation by Shiga-toxin to develop TTP, which supports the “second hit” theory (Desch and Motto, 2007). In real-world research, familial TTP patients with the same ADAMTS13 mutations may have different clinical outcomes, which suggest that isolated ADAMTS13 deficiency is not sufficient to cause TTP (Furlan and Lämmle, 2001; Veyradier et al., 2004).

TTP is mainly induced by infection, pregnancy, autoimmune diseases, drugs, organ transplantation, and cancer (Crawley and Scully, 2013; Kremer Hovinga et al., 2018). Many previous studies have reported an association between HIV and TTP (Benjamin et al., 2009; Masoet et al., 2019). Our patient was a middle-aged man with no history of organ transplantation or autoimmune disease. His tumor markers were negative and radiological imaging was normal. In addition, the patient did not receive drugs that could trigger TTP, such as anti-calcineurin inhibitors, gemcitabine, and vascular endothelial growth factor inhibitors. The screening tests for common viruses, including HBsAg, HCV antibody, syphilis spirochete antibody, CMV IgM, and EBV IgM, were negative. Furthermore, his condition improved without the use of antivirals. The possible factors that may have triggered TTP in our patient included HIV and *S*. Typhimurium infections. Based on the laboratory tests and medical history of the patient, he fulfilled the criteria for AIDS. However, he had no prior episode of TTP before the *S*. Typhimurium infection. In addition, the patient recovered from TTP without ART. Therefore, it is likely that *S*. Typhimurium infection was the main cause of TTP.

We investigated the bacterial triggers of *S*. Typhimurium-induced TTP in this patient. First, we reviewed the literature to identify genes encoding bacterial factors that can trigger TTP or HUS. Then, genes encoding the candidate triggers identified from the literature were searched in the annotation results of *S*. Typhimurium_zhang. We found that intact *norV* gene, which was carried by *S*. Typhimurium_zhang, correlated with increased risk for HUS (Kulasekara et al., 2009). Because *norV* is associated with a high incidence of HUS, its presence could be a trigger of TTP in this patient. The AMR gene analysis identified only two resistance genes, *aac(6’)-Iaa* and *sitABCD*, showing resistance to amikacin, tobramycin, and hydrogen peroxide, which is consistent with the clinical drug sensitivity analysis. This also indicates that TTP in our patient was probably not caused by mutations of AMR genes. Among the virulence factors of *S.* Typhimurium_zhang, *iucC*, and *iucD* were genes encoding biosynthetic enzymes and *iutA* encoded an outer membrane receptor. Production of these three genes plays an important role in synthesis of aerobactin, a citrate-hydroxamate siderophore which enhances the virulence of many pathogens, including *Escherichia, Salmonella, and Shigella* (Di Lorenzo and Stork, 2014; Sheldon et al., 2016). These virulence factors may be related to the severe disease outbreak in this patient. To our knowledge, the relationship between the aerobactin pathway and TTP has not been reported, thus it is worth studying in the future.

We used BLAST to determine the identity of *norV* and its encoded protein FlRd in *S*. Typhimurium_zhang, eight *S*. Typhimurium type strains, and HUS-related *E*. *coli* O157:H7 strain TW14359 (Kulasekara et al., 2009). All eight *S*. Typhimurium type strains carried *norV* gene, and both *norV* gene and FlRd protein had high identity compared to *S*. Typhimurium_zhang and TW14359. Moreover, cTTP cases have previously been reported with *S*. *enterica* infection (Wendt et al., 2021); therefore, the *norV* and FlRd of 73 *S*. *enterica* and *S*. Typhimurium_zhang were also compared, which revealed that *norV* and FlRd in most *S*. *enterica* had high identity with *S*. Typhimurium_zhang. These results indicate that *norV* is possibly linked to high morbidity of patients with TTP.

The phylogenetic analysis revealed that all Enterobacteriaceae carried FlRd and most FlRd in Enterobacteriaceae had a highly conserved amino acid sequence. In the phylogenetic tree, *Salmonella_bongori*_WP_000026020.1 was the closest to, and Hafnia_alvei_WP_025802316.1 and Hafnia_paralvei_WP_130335968.1 were the farthest from, FlRd in *S*. Typhimurium_zhang. Therefore, the three above strains and two reference FlRd proteins were included in the protein alignment along with FlRd in TW14359 and *S*. Typhimurium_zhang. The alignment results revealed that FlRd had constant active sites and crystal structure, indicating a high degree of conservativeness, which suggests that infection by Enterobacteriaceae, with expression of FlRd protein, may be a predisposing factor for TTP. Although the relationships among *norV*, FlRd, and TTP have not been fully elucidated, similar cases require attention in clinical practice.

There were a few limitations to this study. First, the genetic sequencing and analysis were based on a single case. Second, we only analyzed the nucleotide and protein sequences of the bacterial genome. Cohort studies and *in vivo* and *in vitro* experiments, including the use of mouse models, are required to confirm the relationships among *norV*, FlRd, and TTP.

## Conclusion

We report the first case of TTP induced by *S*. Typhimurium in an AIDS patient. Based on the previous literature and our results, FlRd is a potential cause of TTP induced by *S*. Typhimurium. Phylogenetic analysis and protein alignment showed that FlRd encoded by *norV* was functional and highly conserved in Enterobacteriaceae, which suggests that infection by Enterobacteriaceae with expression of FlRd protein may be a risk factor for TTP. Further *in vitro* and *in vivo* research, as well as real-world studies, are required to confirm our results and identify the underlying mechanisms.

## Data Availability Statement

The datasets presented in this study can be found in online repositories. The names of the repository/repositories and accession number(s) can be found below: https://www.ncbi.nlm.nih.gov/genbank/, CP090304.

## Ethics Statement

The studies involving human participants were reviewed and approved by the Ethics Committee of the First Affiliated Hospital, College of Medicine, Zhejiang University (Hangzhou, China). The patients/participants provided their written informed consent to participate in this study. Written informed consent was obtained from the individual(s) for the publication of any potentially identifiable images or data included in this article.

## Author Contributions

YZ and BZ designed the research. ZW, HX, BG, YJ, JM, and TW collected clinical data. ZW, HX, XY, and YL analyzed the data. ZW, HX, BG, YZ, and BZ wrote the manuscript. YZ and BZ reviewed and revised the manuscript. All authors read and approved the final manuscript.

## Funding

This work was supported by National Key R&D Program of China (2021YFC2301900-2021YFC2301901), Key R&D Program of Zhejiang (2022C03125), Independent Task of State Key Laboratory for Diagnosis and Treatment of Infectious Diseases (2021) and the Zhejiang Education Department 2020 Special Project Against COVID-19-Zhejiang University (no.94).

## Conflict of Interest

The authors declare that the research was conducted in the absence of any commercial or financial relationships that could be construed as a potential conflict of interest.

## Publisher’s Note

All claims expressed in this article are solely those of the authors and do not necessarily represent those of their affiliated organizations, or those of the publisher, the editors and the reviewers. Any product that may be evaluated in this article, or claim that may be made by its manufacturer, is not guaranteed or endorsed by the publisher.

## Supplementary Material

Supplementary Figure 1 Amino acid sequence alignment of FlRd According to the result of phylogenetic analysis and searching for template by SWISS-MODEL, 7 FlRd proteins were included in the alignment and 5lmc.2, the protein with highest similarity, were used as crystal structure reference in the alignment. *E. coli*_5lmc.2 and *E. coli*_6etb.1 stands for two most similar FlRd protein with known crystal structure searched by SWISS-MODEL. Amino acid sequence and crystal structure were obtained on Protein Data Base (PDB, https://www.rcsb.org/). *S. bongori*= *Salmonella bongori*_WP_000026020.1 (protein nearest to FlRd of *S.* Typhimurium_zhang on the phylogenetic tree). *H. alvei*= *Hafnia alvei*_WP_025802316.1, *H. paralvei*=*Hafnia paralvei*_WP_130335968.1 (two FlRd protein furthest to FlRd of *S.* Typhimurium_zhang on the phylogenetic tree). *E. coli*_TW14359=FlRd on *E. coli* O157:H7 isolate TW14359. *S.* Typhimurium_zhang=FlRd on *S.* Typhimurium_zhang. Amino acid residues coordinating catalytic iron atoms were marked by * according to previous research (Romão et al., 2016).
