# Supplementary figures and images for: Flavorubredoxin, a Candidate Trigger Related to Thrombotic Thrombocytopenic Purpura: Screening of the Complete Genome of a Salmonella enterica Serovar Typhimurium Isolate From an AIDS Case
Source: Front Cell Infect Microbiol. 2022 Jun 10;12:864087. doi: 10.3389/fcimb.2022.864087 (PMC9226561; doi:10.3389/fcimb.2022.864087)

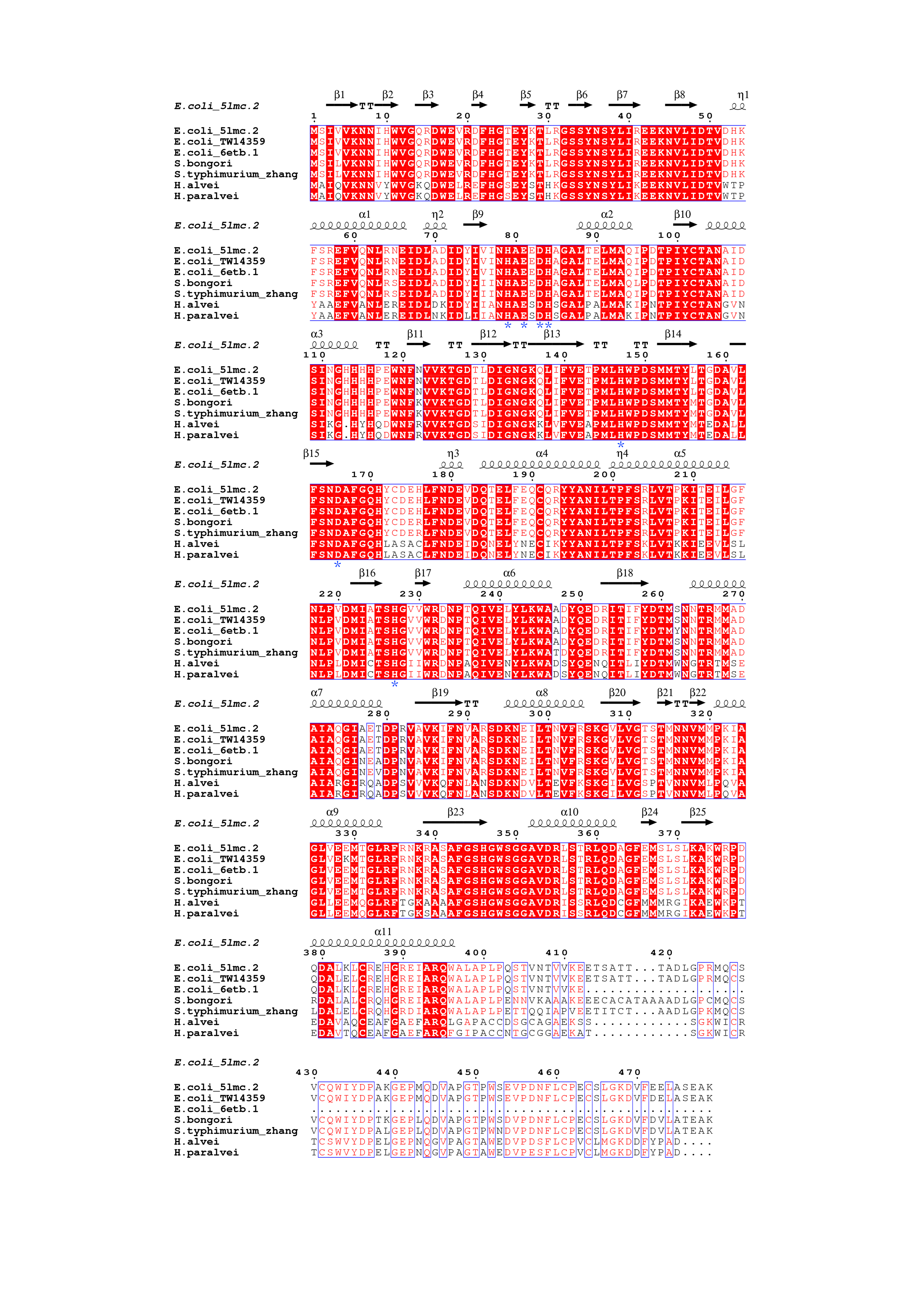

Supplement: Supplementary Figure 1 — Amino acid sequence alignment of FlRd According to the result of phylogenetic analysis and searching for template by SWISS-MODEL, 7 FlRd proteins were included in the alignment and 5lmc.2, the protein with highest similarity, were used as crystal structure reference in the alignment. E. coli_5lmc.2 and E. coli_6etb.1 stands for two most similar FlRd protein with known crystal structure searched by SWISS-MODEL. Amino acid sequence and crystal structure were obtained on Protein Data Base (PDB, https://www.rcsb.org/). S. bongori= Salmonella bongori_WP_000026020.1 (protein nearest to FlRd of S. Typhimurium_zhang on the phylogenetic tree). H. alvei= Hafnia alvei_WP_025802316.1, H. paralvei=Hafnia paralvei_WP_130335968.1 (two FlRd protein furthest to FlRd of S. Typhimurium_zhang on the phylogenetic tree). E. coli_TW14359=FlRd on E. coli O157:H7 isolate TW14359. S. Typhimurium_zhang=FlRd on S. Typhimurium_zhang. Amino acid residues coordinating catalytic iron atoms were marked by * according to previous research (Romão et al., 2016). [file Image_1.tif]
