# Supplementary material for: Flavorubredoxin, a Candidate Trigger Related to Thrombotic Thrombocytopenic Purpura: Screening of the Complete Genome of a Salmonella enterica Serovar Typhimurium Isolate From an AIDS Case
Source: Front Cell Infect Microbiol. 2022 Jun 10;12:864087. doi: 10.3389/fcimb.2022.864087 (PMC9226561; doi:10.3389/fcimb.2022.864087)
Supplement: Supplementary file 3 [file Table_2.docx]

**Supplementary Table 2. Virulence Factors of *S.* Typhimurium_zhang**

| Sequence | Start | End | Strand | Gene | Coverage | Gaps | Coverage (%) | Identity (%) | Accession | Product | Reference |
| --- | --- | --- | --- | --- | --- | --- | --- | --- | --- | --- | --- |
| 1 | 637302 | 639923 | + | bcfC | 1-2622/2622 | 0/0 | 100.00 | 100.00 | NP_459028 | fimbrial usher [bovine colonization factor (Bcf) (AI058)] | [Salmonella enterica subsp. enterica serovar Typhimurium str. LT2] |
| 1 | 1217532 | 1220144 | + | fimD | 1-2613/2613 | 0/0 | 100.00 | 100.00 | NP_459541 | usher protein FimD [Type 1 fimbriae (VF0102)] | [Salmonella enterica subsp. enterica serovar Typhimurium str. LT2] |
| 1 | 2992964 | 2995330 | - | sspH2 | 1-2367/2367 | 0/0 | 100.00 | 100.00 | NP_461184 | type III secretion system effector SspH2 E3 ubiquitin ligase [TTSS(SPI-2 encode) (VF0321)] | [Salmonella enterica subsp. enterica serovar Typhimurium str. LT2] |
| 1 | 3279669 | 3285788 | - | shdA | 1-6120/6120 | 0/0 | 100.00 | 99.97 | NP_461448 | AIDA autotransporter-like protein [ShdA (VF0398)] | [Salmonella enterica subsp. enterica serovar Typhimurium str. LT2] |
| 1 | 3286482 | 3293788 | - | ratB | 1-7308/7308 | 1/1 | 100.00 | 99.99 | NP_461449 | putative outer membrane protein [RatB (VF0399)] | [Salmonella enterica subsp. enterica serovar Typhimurium str. LT2] |
| 1 | 4436736 | 4439264 | - | lpfC | 1-2529/2529 | 0/0 | 100.00 | 99.96 | NP_462539 | long polar fimbrial usher protein LpfC [Lpf (VF0105)] | [Salmonella enterica subsp. enterica serovar Typhimurium str. LT2] |
| 1 | 4567383 | 4570250 | + | misL | 1-2868/2868 | 0/0 | 100.00 | 100.00 | NP_462656 | putative autotransporter [MisL (VF0397)] | [Salmonella enterica subsp. enterica serovar Typhimurium str. LT2] |
| 1 | 4574164 | 4576890 | - | mgtB | 1-2727/2727 | 0/0 | 100.00 | 99.96 | NP_462662 | Mg2+ transport protein [MgtBC (VF0106)] | [Salmonella enterica subsp. enterica serovar Typhimurium str. LT2] |
| 2 | 98472 | 100247 | + | spvB | 1-1776/1776 | 0/0 | 100.00 | 100.00 | NP_490529 | type III secretion system effector SpvB ADP-ribosylation activity [SpvB (VF0107)] | [Salmonella enterica subsp. enterica serovar Typhimurium str. LT2] |
| 2 | 115090 | 117498 | + | pefC | 1-2409/2409 | 0/0 | 100.00 | 100.00 | NP_490509 | plasmid-encoded fimbriae usher protein PefC [Pef (VF0104)] | [Salmonella enterica subsp. enterica serovar Typhimurium str. LT2] |
| 2 | 134713 | 137014 | - | iutA | 1-2280/2280 | 3/24 | 101.01 | 88.36 | NP_755498 | ferric aerobactin receptor precusor IutA [Aerobactin (VF0229)] | [Escherichia coli CFT073] |
| 2 | 138270 | 140012 | - | iucC | 1-1743/1743 | 0/0 | 100.00 | 96.04 | NP_755500 | aerobactin siderophore biosynthesis protein IucC [Aerobactin (VF0229)] | [Escherichia coli CFT073] |
| 2 | 140960 | 142747 | - | iucA | 1-1788/1788 | 0/0 | 100.00 | 94.18 | NP_755502 | aerobactin siderophore biosynthesis protein IucD [Aerobactin (VF0229)] | [Escherichia coli CFT073] |
