# Supplementary material for: Flavorubredoxin, a Candidate Trigger Related to Thrombotic Thrombocytopenic Purpura: Screening of the Complete Genome of a Salmonella enterica Serovar Typhimurium Isolate From an AIDS Case
Source: Front Cell Infect Microbiol. 2022 Jun 10;12:864087. doi: 10.3389/fcimb.2022.864087 (PMC9226561; doi:10.3389/fcimb.2022.864087)
Supplement: Supplementary file 4 [file Table_3.docx]

**Supplementary Table 3. Antimicrobial resistance genes of *S.* Typhimurium_zhang**

| Sequence | Start | End | Strand | Gene | Coverage | Gaps | Coverage (%) | Identity (%) | Accession | Product | Resistance |
| --- | --- | --- | --- | --- | --- | --- | --- | --- | --- | --- | --- |
| 1 | 2317864 | 2318301 | + | aac(6')-Iaa_1 | 1-438/438 | 0/0 | 100.00 | 99.77 | NC_003197 | aac(6')-Iaa | Amikacin;Tobramycin |
| 2 | 146016 | 149465 | - | sitABCD_1 | 1-3459/3459 | 3/9 | 100.00 | 99.65 | AY598030 | sitABCD | Hydrogen_peroxide |
